# *Rhodotorula mucilaginosa* Fungemia in an Infected Biloma Patient Following a Traumatic Liver Injury

**DOI:** 10.3390/healthcare12090880

**Published:** 2024-04-24

**Authors:** Mohammad Nizam Mokhtar, Raha Abdul Rahman, Farah Hanim Abdullah, Izzuddin Azaharuddin, Azarinah Izaham, Chuan Hun Ding

**Affiliations:** 1 Department of Anaesthesiology & Intensive Care, Faculty of Medicine, Universiti Kebangsaan Malaysia, Kuala Lumpur 56000, Malaysia; raha@ukm.edu.my (R.A.R.); farahha0301@gmail.com (F.H.A.); izzuddin@ukm.edu.my (I.A.); azaizaham@yahoo.com (A.I.); 2 Department of Medical Microbiology & Immunology, Faculty of Medicine, Universiti Kebangsaan Malaysia, Kuala Lumpur 56000, Malaysia; dingch@ppukm.ukm.edu.my

**Keywords:** biloma, *Rhodotorula mucilaginosa*, fungemia, liver, trauma

## Abstract

*Rhodotorula mucilaginosa* fungemia is rare and highly resistant to antifungal therapy. We herein report a case involving a 31-year-old male admitted after a high-velocity road traffic accident. He sustained a grade IV liver injury with right hepatic vein thrombosis, which necessitated an urgent laparotomy. Post-operatively, repeated imaging of the abdomen revealed the presence of a biloma. Percutaneous subdiaphragmatic drainage was carried out but appeared ineffective, prompting a second surgery for an urgent hemi-hepatectomy. The patient was then nursed in the intensive care unit (ICU); however, during his stay in the ICU, he became more sepsis, which was evident by worsening ventilatory support and a rise in septic parameters from the biochemistry parameters. Despite intravenous piperacillin–tazobactam and fluconazole, his septic parameters did not improve and a full septic workup was conducted and was found to be positive for *Rhodotorula mucilaginosa* from the blood cultures. After discussion with the infectious disease physicians and clinical microbiologists, it was decided to initiate a course of intravenous meropenem and amphotericin B based on minimum inhibitory concentration (MIC) values, considering the patient’s extended ICU stay and catheter use. Eventually, after successfully weaning off mechanical ventilation, the patient was discharged from ICU care. This case underscores the necessity of individualized approaches, combining timely imaging, appropriate drainage techniques, and tailored treatments to optimize outcomes for such intricate post-traumatic complications.

## 1. Introduction

*Rhodotorula* is a yeast-like fungus commonly found in various environmental niches, including soil, water, air, and plants. They are considered to be commensal fungi belonging to the family of Sporidiobolaceae, with *R. mucilaginosa*, *R. glutinis*, and *R. minuta* typically being the most clinically encountered species, especially in the Asia Pacific region [1]. However, in an immunocompromised patient, these fungi can develop into opportunistic infections, leading to fungemia, pneumonia, endocarditis, and meningitis. *Rhodotorula* species infections can be directly transmitted via airborne spores or droplets, enteral ingestion of contaminated food or water, and cross-infection from individuals or contaminated surfaces. Additionally, invasive medical procedures, which may include surgery and long-term indwelling catheterization, can introduce the pathogen into the body, leading to nosocomial infections. *Rhodotorula* infections have a 10% mortality rate and are commonly associated with profound sepsis and shock with multiorgan involvement, which is attributed to the high resistance profile of the organism towards our conventional antifungal agents [2,3]. Thus, it is crucial for a timely clinical assessment and detection of *Rhodotorula mucilaginosa* infections with prompt initiation of an adequately dosed targeted antifungal therapy to prevent further resistance. We report a case of a traumatic patient who developed *Rhodotorula mucilaginosa* fungemia in an immunocompetent host who received early antifungal selection to prevent potential morbidity and mortality associated with it.

## 2. Detailed Case Description

We present a case involving a 31-year-old male who was admitted following a high-velocity road traffic accident. Upon assessment in the emergency department, he was intubated due to respiratory compromise identified during the primary survey. His injuries included multiple segmental rib fractures with a right-sided hemopneumothorax, a right-sided Schatzker’s type IV tibial plateau fracture, a grade IV liver injury with right hepatic vein thrombosis, and hemoperitoneum. He was then transferred to the intensive care unit (ICU) for further management.

Subsequently, within 24 h of admission to the ICU, he developed abdominal compartment syndrome, manifesting signs of hemodynamic compromise. A contrast-enhanced computed tomography (CECT) of the abdomen revealed gross hemoperitoneum requiring an urgent laparotomy. During the surgery, bleeding from segment VI of the liver was identified, and surgical hemostasis with preservation of the liver parenchyma was performed by the hepatobiliary surgeon, with an estimated blood loss of 2.2 L, and the patient was then nursed in the ICU post-operatively.

**Figure 1:**
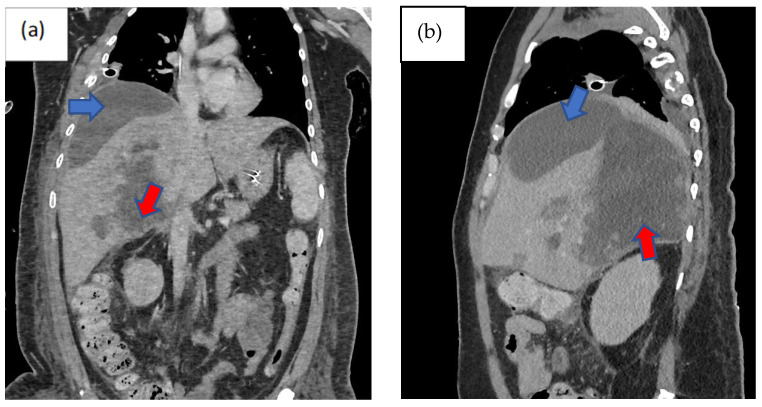

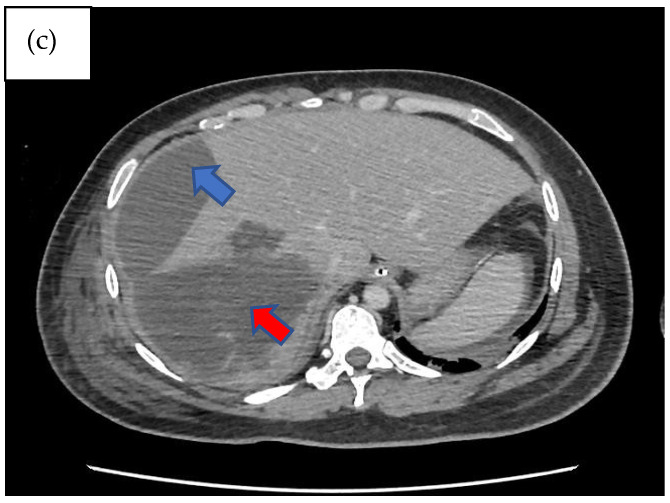
Computed tomography scan of the abdomen non-contrasted phase showing heterogenous areas consisting of hematoma and laceration at segments V, VI, VII, and VIII extending down to the subhepatic region (red arrow). Inferiorly, it extends down to the hepatic flexure, just lateral to the right pararenal space. Another subcapsular collection was seen adjacent to right liver lobe (blue arrow): (**a**) coronal view, (**b**) sagittal view, and (**c**) axial view, with findings as aforementioned.

Three days following the laparotomy, the patient developed ventilator-associated pneumonia and intra-abdominal sepsis and was treated empirically with fluconazole and piperacillin–tazobactam. Repeated imaging of the abdomen was carried out in view of a poor response to antimicrobial therapy and showed the formation of biloma (Figure 1). A percutaneous subdiaphragmatic drain was inserted under computed tomography (CT) guidance by the interventional radiology team to facilitate drainage, despite being able to drain only a minimal amount, and the drain was dislodged after three days. In view of ineffective percutaneous drainage with worsening intrabdominal sepsis despite one week of adequate antimicrobial therapy, upon discussion with the hepatobiliary team, we decided to proceed with a hemi-hepatectomy for the removal of the biloma.

**Figure 2:**
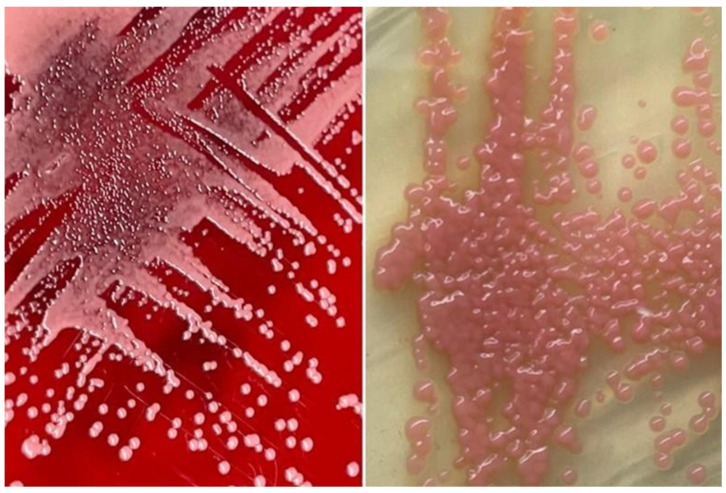
Salmon-pink colonies of *Rhodotorula mucilaginosa* on sheep blood agar (**left**) and on Sabouraud dextrose agar (**right**). Sheep blood agar was initially used as it is the standard isolation media used for all positive blood cultured in the bacteriology laboratory. Once the organism was found to be yeast, a subculture onto mycological isolation media such as Sabouraud dextrose agar was carried out.

**Figure 3:**
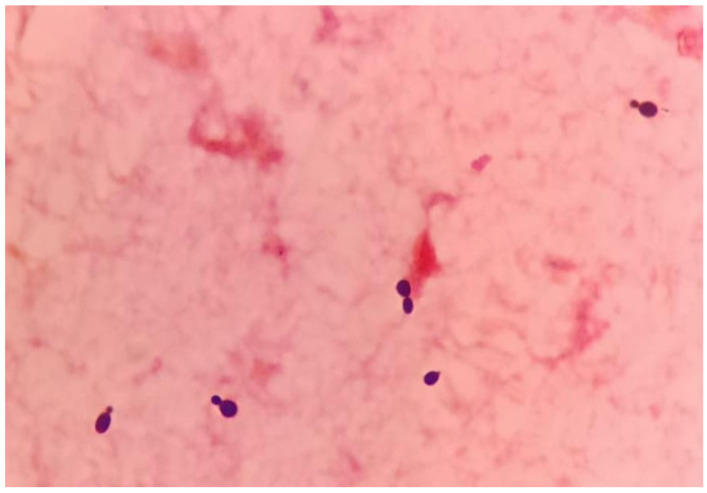
A Gram stain of the positive blood culture showing budding yeast cells (1000× magnification). The yeast was identified as *Rhodotorula mucilaginosa* (%ID: 91.9) through biochemical means using the ID 32 C kit (Biomerieux, Marcy-l’Étoile, France). Our isolate’s identity was further confirmed by matrix-assisted laser desorption ionization–time of flight mass spectrometry (MALDI Biotyper, Bruker-Daltonics, Bremen, Germany), which matched its mass spectral pattern with that of *Rhodotorula mucilaginosa* DSM 70403 DSM.

Following urgent adequate source control via hemi-hepatectomy and biloma removal along with empirical antimicrobial coverage, the patient remained hemodynamically unstable, requiring vasopressors. Septic parameters including procalcitonin were persistently elevated, pointing towards unresolved infection states. Repeated blood culture and sensitivity testing for both bacterial and fungal specimens, which were sent pre-operatively, further identified and reported as salmon-pink colonies, and an early clue of the presence of yeast and subculture onto mycological isolation media revealed that the growth was *Rhodotorula mucilaginosa* (Figure 2 and Figure 3).

After a multidisciplinary discussion involving infectious diseases physicians and clinical microbiologists, it was concluded based on the identification and minimum inhibitory concentration (MIC) testing to initiate a course of intravenous meropenem and amphotericin B. This decision was supported by the multiple risk factors including immunocompromised states and prolonged hospital stay, especially in a critical care setting with multiple indwelling catheters with persistent signs of sepsis. MIC values are shown in Table 1.

Following treatment, the patient’s condition improved significantly, requiring less ventilatory support and showing notable decreases in inflammatory markers. After a week of intravenous meropenem and amphotericin B, successful liberation from mechanical ventilation occurred, leading to discharge from the ICU after 21 days. Continued intravenous therapy in the surgical ward completed a 6-week regimen advised by the infectious disease physician.

## 3. Discussion

Biloma is a rare abnormal bilious collection of either intrahepatic or extrahepatic region due to bacterial colonization of bile as a result of trauma or biliary diseases. Biloma as a result of trauma can take 1 to 2 days to appear [4]. They have the propensity to become infected due to microbial ascent, bacteremia, or catheter colonization and may lead to serious and life-threatening complications such as peritonitis, biliopleural fistula, and hemobilia [5]. Approximately 16% of cases are associated with bacteremia at the time of biloma diagnosis [6]. Fungal infections associated with bilomas are uncommon, with liver transplantation accounting for the majority, presenting an incidence of up to 26% [7]. The clinical presentation of biloma varies as it may present as diffused or localized abdominal pain, jaundice, fever, and peritonitis [5]. However, in this unique case, the patient, an immunocompetent host, developed fungemia associated with a biloma, marking the novelty of this presentation. The deviation from the typical association with liver transplantation underscores the exceptional nature of this particular clinical scenario.

Imaging remains the gold standard for diagnosing bilomas, with ultrasound (US) often being the initial imaging modality used. Bilomas typically appear as well-defined hypoechoic fluid collections within the abdomen that vary in size and shape, ranging from small localized areas to larger, more extensive masses. It also has the capability of detecting the content within the biloma by showing a variety of findings ranging from well-defined collections in the liver parenchyma to extensive fluid collections across the abdomen [8]. Notably, US findings show heavily loculated bilomas are often linked to infection [9]. CT imaging can help identify the overall structure of the biloma, delineating clear margins that can be either encapsulated or non-encapsulated [10]. While CT imaging offers a more intricate view of bilomas, it lacks the capability to conclusively distinguish among various potential diagnoses, including seroma, abscess, lymphocele, liver cyst, hematoma, and pseudocyst [11]. Consequently, additional imaging modalities such as magnetic resonance (MR) imaging or hepatobiliary cholescintigraphy may be necessary to help validate the diagnosis. Direct sampling of the biloma together with these imaging techniques will also help with the diagnosis [10]. In T1-weighted images, bilomas will display low signal intensity, whereas in T2-weighted images, high signal intensity is seen [12]. Furthermore, MR imaging can help to define the characteristics of a biloma. While contrast infrequently penetrates the biloma, rim enhancement and septations occasionally occur due to reactive inflammation and infection [12]. Hepatobiliary cholescintigraphy serves as a highly efficient non-invasive imaging technique for diagnosing and strategizing treatment for bilomas by utilizing a radiotracer known as Tc-99m iminodiacetic acid, which is commonly referred to as hepatobiliary iminodiacetic acid (HIDA) imaging [10,13]. HIDA imaging exhibits a high level of sensitivity in detecting bile leaks [13]. Nonetheless, it lacks the ability to offer detailed imaging of the surrounding anatomical structures. Single positron emission computed tomography (SPECT) is another imaging technique that has the ability to furnish more intricate imaging of potential leak locations, proving particularly valuable in planning percutaneous image-guided drains [10]. Endoscopic retrograde cholangiopancreatography (ERCP) and percutaneous transhepatic cholangiogram (PTC) can be considered not only for diagnostic tests but also may offer some degree of intervention either percutaneously or endoscopically [13]. Other minimally invasive investigations available are CT-guided sampling technique and US-guided sampling technique. However, these techniques require laboratory assistance to analyse the sample collected [10]. In our case, CT imaging was sufficient to give us the information we need. The initial CT imaging (Figure 1) revealed a rim-enhancing lesion surrounding the biloma, suggesting a potential infective biloma. However, we did not perform a biopsy and or staining hepatic tissue during the hemi-hepatectomy to definitely confirm that *Rhodotorula mucilaginosa* originated from the biloma itself. This limitation underscores a key aspect of our study, highlighting the need for further investigations to establish a direct link between the presence of *Rhodotorula mucilaginosa* and the biloma. MR imaging was not performed in this patient which shows another limitation in this case. MR imaging might indicate presence of septations within the biloma and this might be the reason why percutaneous drainage failed in this patient.

The management of bilomas involves a combination of antibiotic treatment and drainage of the bilomas concurrently. Antibiotic therapy should be initiated empirically based on likely pathogens and the patient’s clinical condition, with the choice guided by culture and sensitivity reports. In our case, *Rhodotorula mucilaginosa* was isolated from the blood culture, and we posit that its source may have originated either from the biloma, possibly due to environmental contamination during the traumatic event, or through opportunistic infection from commensal sites. It is important to ensure that the bilomas are drained either percutaneously, endoscopically, or surgically, depending on the location and size, as antibiotic treatments may not effectively penetrate the layers of tissue surrounding the biloma or reach deep-seated pockets of infection [8].

*Rhodotorula*, a yeast often mistaken for Candida species due to its similar morphology, is a low-virulence organism widely distributed in the environment [2]. Distinguishing between *Candida* and *Rhodotorula* sp. based on gram stain can pose considerable challenges, as both exhibit oval budding yeast with rudimentary pseudohyphae [14]. Utilizing the positive urease test as a quick pointer to differentiate between the *Rhodotorula* sp. and *Candida* sp. can be advocated but it is important to acknowledge the limitations of this approach, including the test’s positive yield with certain *Candida* sp. [15]. Laboratories with limited expertise in mycology might lead to delayed treatment with amphotericin B due to its similar morphology. Fortunately, in this case, we started the treatment early and did not adversely affect the outcome of the patient. Though previously considered non-pathogenic, *Rhodotorula* sp. has emerged as an opportunistic etiologic agent, particularly in cases of fungemia associated with catheters, endocarditis, peritonitis, meningitis, and endophthalmitis [8]. In a recent review, *Rhodotorula* fungemia accounted for 48.6% of all *Rhodotorula*-related infections, with *Rhodotorula mucilaginosa* identified as the predominant causative species [2]. *Rhodotorula* fungemia has been associated with a crude mortality rate of up to 20%, especially in patients with indwelling vascular catheters, granulocytopenia, impaired anatomic barriers, cellular immune dysfunction, and parenteral nutrition [16]. Isolation of *Rhodotorula* sp. from sterile sites such as blood, peritoneal fluid, or cerebrospinal fluid suggests infection, unlike non-sterile sites like skin, sputum, or stool, as demonstrated in our case report [17]. Despite lacking apparent immunosuppression, our patient possessed multiple risk factors predisposing him to invasive fungal infections, including ventilator-associated pneumonia, broad-spectrum antimicrobial therapy, admission to the critical care unit with CVC access, and urinary catheterization, all significant contributors to invasive fungal infection. Persistent sepsis despite biloma removal and administration of potent broad-spectrum antibiotics raised suspicion of fungal infection. Therefore, clinical judgment dictated coverage with Amphotericin B and meropenem to address the fungal infection.

Managing bilomas in combination with *Rhodotorula mucilaginosa* fungemia poses a challenge. This necessitates a multidisciplinary approach involving infectious disease physicians, interventional radiologists, and surgeons. In the past, surgery was the only option for treating biloma but with the advancement of medical technology, more options are currently available [5]. The optimal approach to treating bilomas hinges on several factors, including the patient’s overall health, the size and location of the biloma, the presence of biliary leakage, and the occurrence of sepsis [18]. Small biloma does not necessitate any treatment and close observation suffices [5]. Percutaneous drainage, the most common method, is associated with lower morbidity and mortality compared to surgical drainage [19]. In our case, the patient initially underwent CT-guided drainage of the biloma. Unfortunately, our initial attempt at percutaneous drainage proved unsuccessful, likely due to the presence of multiple septated collections within the biloma. While additional drainage procedures could be considered, introducing more drains poses an increased risk of infection for the patient. Endoscopic drainage was not suitable for our case as it requires large collections and those located in the left lobe of the liver. Eventually, due to persistent sepsis, our patient necessitated a hemi-hepatectomy for source control, underscoring the role of surgical intervention in cases where percutaneous drainage fails or bile leaks persist.

In vitro studies have consistently shown that *Rhodotorula* species, including *Rhodotorula mucilaginosa*, display resistance to fluconazole and echinocandins. However, they are more susceptible to other azoles such as voriconazole and itraconazole [20,21,22]. In the clinical setting, the high resistance of *Rhodotorula* species to fluconazole poses a significant challenge in selecting appropriate antifungal therapy. As a result, alternative agents with demonstrated efficacy against *Rhodotorula*, such as voriconazole, caspofungin, and amphotericin B, are often considered. Among these alternatives, amphotericin B has shown consistent activity against *Rhodotorula* species, making it a viable option for treatment. One key property of amphotericin B is its mechanism of action, which involves binding to ergosterol, a crucial component of fungal cell membranes [23]. This binding disrupts the integrity of the cell membrane, leading to leakage of intracellular contents and, ultimately, cell death [23]. In our case, concentration gradient tests were conducted, revealing a low minimum inhibitory concentration (MIC) for amphotericin B, indicating susceptibility. However, the other antifungals, azoles (fluconazole, itraconazole, posaconazole, voriconazole), and echinocandins (anidulafungin, caspofungin) showed high MICs, indicating poor response to treatment (Table 1). Comparing our findings with those reported in the literature, similar MIC values for amphotericin B were observed in another case of *Rhodotorula mucilaginosa* fungemia with MIC of 0.25 μg/mL and 128 μg/mL for amphotericin B and fluconazole, respectively [24]. Clinically, this was evident when the patient’s condition did not improve despite adequate dosing and duration of fluconazole during the initial treatment.

In addition to the patient’s clinical response and infection resolution, several other factors contribute to determining the duration of antifungal therapy. These factors include the severity of the infection, the presence of underlying medical conditions, such as diabetes or immunosuppression, and the risk of recurrence. Close monitoring of the patient’s symptoms, laboratory results, and imaging findings is essential in assessing the effectiveness of the antifungal treatment and guiding the decision regarding treatment duration. At present, there is no established standard duration for antifungal therapy in patients with *Rhodotorula mucilaginosa* fungemia, highlighting a potential area for future research aimed at establishing optimal treatment durations. In our case, the decision to administer a total of six weeks of targeted antifungal therapy from the point of diagnosis was based on a thorough evaluation of the factors mentioned. Regular follow-up examinations and imaging studies were conducted to track the progress of the treatment and ensure the resolution of the infection. Regarding the role of prophylactic antifungal therapy in patients with infected biloma, while some studies suggest its potential benefits in reducing fungal infections, especially in immunocompromised individuals, controversy exists regarding its routine use [25,26,27]. Prophylactic antifungal therapy may carry risks, such as the development of antifungal resistance and adverse drug reactions, which need to be weighed against its potential benefits [28]. Therefore, its use should be carefully considered on a case-by-case basis, with preference given to high-risk patients, such as those undergoing extensive surgical procedures or with significant immunosuppression [27]. Close collaboration between infectious disease specialists, hepatobiliary surgeons, and other relevant healthcare providers is crucial in making informed decisions regarding prophylactic antifungal therapy in this patient population.

The prognosis of infected biloma combined with *Rhodotorula mucilaginosa* fungemia is generally favorable if diagnosed early and managed appropriately. However, delayed diagnosis or inappropriate management can lead to severe morbidity and mortality, which ranges from 30% to 50% mortality [17]. Factors associated with poor outcomes include delays in initiating appropriate antifungal therapy, sepsis, and underlying comorbidities such as liver disease [28]. In addition to the factors mentioned, the presence of *Rhodotorula mucilaginosa* fungemia may also increase the risk of disseminated infection, potentially affecting multiple organ systems and complicating treatment. Therefore, early recognition of systemic involvement and prompt initiation of systemic antifungal therapy are crucial to prevent disease progression and improve prognosis [10]. Furthermore, the immune status of the patient plays a significant role in determining outcomes, with immunocompromised individuals facing a higher risk of severe complications and mortality. As such, efforts to optimize immune function through supportive measures or targeted interventions may contribute to better treatment responses and enhanced survival rates. Overall, a comprehensive understanding of the multifactorial nature of infected biloma with *Rhodotorula mucilaginosa* fungemia is essential for devising effective management strategies and improving patient outcomes.

## 4. Conclusions

Further investigation into the optimal duration of antimicrobial therapy, particularly for fungal infections like *Rhodotorula mucilaginosa*, could be valuable. Moreover, advancements in diagnostic modalities for detecting and monitoring fungal infections, such as *Rhodotorula mucilaginosa* fungemia, could lead to earlier and more accurate diagnoses. Potential future research could aim to determine whether a shorter or longer course of therapy is more effective in achieving complete eradication of the infection, while minimizing the risk of resistance and the development of rapid, sensitive, and specific diagnostic tests, including molecular assays or biomarker-based approaches. In conclusion, managing post-traumatic liver bilomas when complicated with rare infections such as *Rhodotorula mucilaginosa* fungemia can be very complex and challenging. This case is a good example that, with appropriate early imaging for diagnosis, multidisciplinary collaboration with timely drainage procedures, either through percutaneous or open surgery and targeted antimicrobial therapy, is pivotal in managing such cases effectively.

## Figures and Tables

**Table 1:** Antifungal minimal inhibitory concentration (MIC) values for *Rhodotorula mucilaginosa.* Antifungal susceptibility testing was performed using the broth microdilution kit Sensititre YeastOne YO10 (TREK Diagnostic Systems, Independence, OH, USA).

Antifungal Agent	Minimal Inhibitory Concentration (μg/mL)
Amphotericin B	0.25
Fluconazole	128
Itraconazole	1
Voriconazole	2
Posaconazole	1
Anidulafungin	≥8
Caspofungin	≥8
Micafungin	≥8
Flucytosine	0.06

## Data Availability

Data are contained within the article.
